# Individualized music induces theta-gamma phase-amplitude coupling in patients with disorders of consciousness

**DOI:** 10.3389/fnins.2024.1395627

**Published:** 2024-07-01

**Authors:** Qiuyi Xiao, Xiaochun Zheng, Yun Wen, Zhanxing Yuan, Zerong Chen, Yue Lan, Shuiyan Li, Xiyan Huang, Haili Zhong, Chengwei Xu, Chang’an Zhan, Jiahui Pan, Qiuyou Xie

**Affiliations:** ^1^Joint Research Centre for Disorders of Consciousness, Department of Rehabilitation Medicine, Zhujiang Hospital, Southern Medical University, Guangzhou, Guangdong, China; ^2^School of Laboratory Medicine and Biotechnology, Southern Medical University, Guangzhou, Guangdong, China; ^3^Music and Reflection Incorporated, Guangzhou, Guangdong, China; ^4^Guangdong Provincial Key Laboratory of Medical Image Processing, Southern Medical University, Guangzhou, Guangdong, China; ^5^School of Software, South China Normal University, Guangzhou, Guangdong, China; ^6^Department of Hyperbaric Oxygen, Zhujiang Hospital, Southern Medical University, Guangzhou, Guangdong, China; ^7^School of Rehabilitation Sciences, Southern Medical University, Guangzhou, Guangdong, China

**Keywords:** disorders of consciousness, music, electroencephalography, phase-amplitude coupling, cross-modal influences

## Abstract

**Objective:**

This study aimed to determine whether patients with disorders of consciousness (DoC) could experience neural entrainment to individualized music, which explored the cross-modal influences of music on patients with DoC through phase-amplitude coupling (PAC). Furthermore, the study assessed the efficacy of individualized music or preferred music (PM) versus relaxing music (RM) in impacting patient outcomes, and examined the role of cross-modal influences in determining these outcomes.

**Methods:**

Thirty-two patients with DoC [17 with vegetative state/unresponsive wakefulness syndrome (*VS*/UWS) and 15 with minimally conscious state (MCS)], alongside 16 healthy controls (HCs), were recruited for this study. Neural activities in the frontal–parietal network were recorded using scalp electroencephalography (EEG) during baseline (BL), RM and PM. Cerebral-acoustic coherence (CACoh) was explored to investigate participants’ abilitiy to track music, meanwhile, the phase-amplitude coupling (PAC) was utilized to evaluate the cross-modal influences of music. Three months post-intervention, the outcomes of patients with DoC were followed up using the Coma Recovery Scale-Revised (CRS-R).

**Results:**

HCs and patients with MCS showed higher CACoh compared to *VS*/UWS patients within musical pulse frequency (*p* = 0.016, *p* = 0.045; *p* < 0.001, *p* = 0.048, for RM and PM, respectively, following Bonferroni correction). Only theta-gamma PAC demonstrated a significant interaction effect between groups and music conditions (*F*_(2,44)_ = 2.685, *p* = 0.036). For HCs, the theta-gamma PAC in the frontal–parietal network was stronger in the PM condition compared to the RM (*p* = 0.016) and BL condition (*p* < 0.001). For patients with MCS, the theta-gamma PAC was stronger in the PM than in the BL (*p* = 0.040), while no difference was observed among the three music conditions in patients with *VS*/UWS. Additionally, we found that MCS patients who showed improved outcomes after 3 months exhibited evident neural responses to preferred music (*p* = 0.019). Furthermore, the ratio of theta-gamma coupling changes in PM relative to BL could predict clinical outcomes in MCS patients (*r* = 0.992, *p* < 0.001).

**Conclusion:**

Individualized music may serve as a potential therapeutic method for patients with DoC through cross-modal influences, which rely on enhanced theta-gamma PAC within the consciousness-related network.

## 1 Introduction

Prolonged disorders of consciousness (DoC), resulting from severe brain injuries, encompass a continuum of conditions ranging from vegetative state/unresponsive wakefulness syndrome (*VS*/UWS) (Laureys et al., 2010; Giacino et al., 2018) to minimally conscious state (MCS) (Giacino et al., 2002). *VS*/UWS is a complex neurological condition in which patients appear to be awake but show no sign of awareness of themselves or their environment, exhibiting only reflexive, non-purposeful behavior. Conversely, MCS patients not only appear wakeful but also exhibit inconsistent but reproducible signs of awareness, such as non-reflexive behavior to external stimuli. The clinical management of patients with DoC remains challenging due to insufficient empirical evidence supporting various therapeutic approaches.

Music, as a form of sensory stimulation, holds promise as a valuable intervention for patients with DoC. Its emotionally salient characteristics engage various brain regions, including the auditory cortices, the sensorimotor network, and the limbic systems (Chen et al., 2022; Vuust et al., 2022). In addition, music possesses a self-referential characteristic. It has been contented that personally relevant music (i.e., individualized music or preferred music) captures attention more easily (Mack et al., 2002) and elicits a more pronounced cerebral response in patients with DoC (Heine et al., 2015, 2017; Magliacano et al., 2019; Martínez-Molina et al., 2021). Meanwhile, a previous study demonstrated that relaxing music could induce relaxation and enhance functional connectivity in theta and alpha bands among health subjects, suggesting that relaxing music could facilitate both relaxation and alertness in participants (Mahmood et al., 2022). Additionally, relaxing music has the potential to reinforce positive behaviors and mitigate undesired behaviors following brain injury (Bower et al., 2014). Therefore, we compared these two types of music to determine which was more suitable for patients with DoC.

At present, the mechanism underlying the effect of music on the brain remains elusive. It has been argued that music, as an auditory modality stimulation, may affect the neural processing of another modality, such as consciousness, which is called cross-modal influences (Bauer et al., 2020). The Global Workspace Theory (GWT) posits that consciousness relies on ignition and broadcast occurring within a neuronal global workspace, where the frontal–parietal network assumes a central role as a hub-like entity (Seth and Bayne, 2022). The frontal–parietal network consists of the default mode network (DMN) and the executive control network (ECN), both of which are involved in awareness (Vanhaudenhuyse et al., 2011). Therefore, the consciousness-related network delineated herein refers to the frontal–parietal network (Modolo et al., 2020; Edlow et al., 2021). Cross-modal influences in cortices are regulated by the synchronization of ongoing neural oscillations, measurable using electroencephalography (EEG). Neural entrainment may provide the basis for synchronization of oscillatory activity, which refers to the phenomenon where intrinsic neural oscillations gradually become phase-locked to external rhythmic stimuli, aligning the temporal dynamics of neural processing to external rhythms (Lakatos et al., 2008). Music, a natural rhythmic stimulus, consists of two main rhythmic elements: beat (pulse) and meter. The beat can be grouped or subdivided in meter, corresponding to harmonics or sub-harmonics of the pulse frequency. Musical rhythms occur in a specific frequency range that corresponds to the delta and theta frequency bands (London, 2004; Large et al., 2015). Musical rhythms stimulate the production of low-frequency brain rhythms, such as delta and theta waves, through neural entrainment, providing a basis for the perception of musical rhythms (Nozaradan et al., 2012; Doelling and Poeppel, 2015; Weineck et al., 2022). Meanwhile, high-frequency brain activities are thought to reflect elaborate processing of music (Fujioka et al., 2009; Arnal and Giraud, 2012; Doelling and Poeppel, 2015; Herff et al., 2020). Handling music necessitates the engagement of diverse brain oscillations.

Cross-frequency coupling (CFC), integrating different rhythmic neural oscillations, has been demonstrated to serve as a mechanism for synchronization and communication in the brain (Canolty and Knight, 2010; González et al., 2020). Phase-amplitude coupling (PAC), a subset of CFC, is essential for processing sensory and cognitive information (Canolty et al., 2006; Abubaker et al., 2021; Grover et al., 2021; Riddle, 2021). A previous study found that auditory stimulation evoked PAC not only within the olfactory cortex but also between the auditory cortex and olfactory cortex. Stronger cross-regional PAC occurred during correct trials, suggesting that phase-amplitude coupling participated in cross-modal sensory information processing (Zhou et al., 2019). However, whether music impacts on the PAC of patients with DoC is unknown as there are no related reports.

In this study, we observed distinct frequency pairs of PAC using scalp EEG during baseline (BL), relaxing music (RM) and preferred music (PM) conditions. This study had three main objectives. First, we aimed to detect whether patients with DoC could track to music. Second, we investigated the cross-modal influences of music on patients with DoC through PAC with the EEG data from frontal–parietal region. Third, we examined whether individualized music is more effective compared to relaxing music and whether these cross-modal influences could determine patient’s outcomes. We hypothesized that patients with DoC could track to music and that preferred music could enhance PAC within the consciousness-related network, thereby promoting brain connection, which may serve as a mechanism for music-promoted consciousness restoration. Due to the personal peculiarity, preferred music is expected to elicit more pronounced coupling in comparison to relaxing music. Stronger coupling is anticipated to be associated with better prognosis, providing a basis for selecting appropriate music for patients with DoC.

## 2 Materials and methods

### 2.1 Participants

This study recruited thirty-two patients with DoC (17 *VS*/UWS, 15 MCS). The following were the inclusion criteria: (1) age between 18 and 70-year-old; (2) *VS*/UWS or MCS diagnosed by Coma Recovery Scale-Revised (CRS-R) (Giacino et al., 2004); (3) time since injury between 28 days and 1 year; and (4) auditory subscale of CRS-R ≥ 1 or absence of auditory injury at least one side, as determined by Brainstem Auditory Evoked Potentials (BAEP). The following were the exclusion criteria: (1) epilepsy; and (2) the use of sedative drugs within the previous 24 h before EEG recording. The CRS-R assessment was performed by two trained staff members prior to the study, with a follow-up CRS-R assessment administered to assess outcomes 3 months later. One patient (*VS*/UWS) was excluded because of low signal-to-noise ratio (SNR) EEG data. Therefore, 31 patients were included in the phase-amplitude coupling analysis. However, due to incomplete audio recordings from one *VS*/UWS patient and one MCS patient, we included fifteen *VS*/UWS patients and fourteen MCS patients in the cerebral-acoustic coherence analysis. Additionally, we divided patients with DoC into two groups according to their outcomes at 3 months. Patients whose consciousness level had improved were considered to have positive outcomes, while those whose consciousness level had not changed or decreased were considered to have negative outcomes. So MCS patients with positive outcomes were regarded as the positive group (MCS_P), while MCS with negative outcomes were regarded as the negative group (MCS_N). *VS*/UWS patients with positive outcomes were regarded as the positive group (*VS*/UWS_P), while *VS*/UWS with negative outcomes were regarded as the negative group (*VS*/UWS_N).

Sixteen healthy controls (HCs) were also enrolled in this study, with the following inclusion criteria: (1) no neurological history; (2) normal hearing; and (3) no intemperance. The study was approved by the ethics committee of Zhujiang Hospital and registered as NCT05382260 in clinical trials. All HCs and legally authorized representatives of patients provided written informed consent.

### 2.2 Study procedure

**Figure 1:**
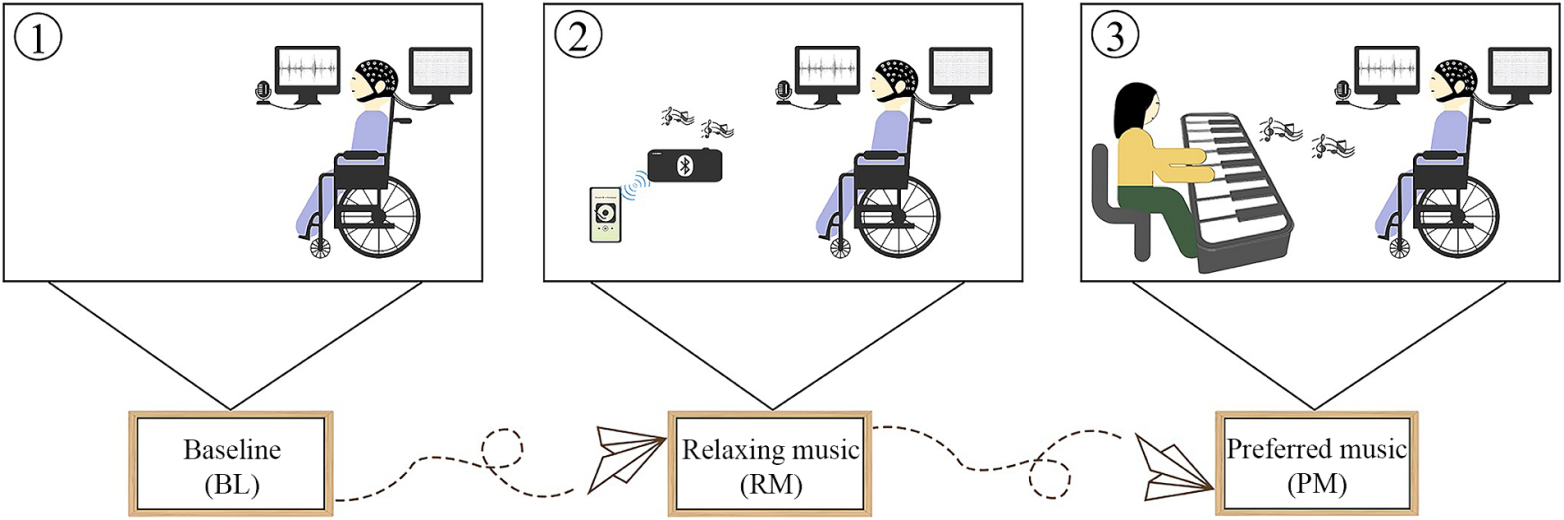
The flow chart of this research.

Following a 5-min BL, participants listened to two different types of music: relaxing music and preferred music. To minimize order effects, the order of music was randomly performed with at least 3 min washout separating them. The flow chart of our research is shown in Figure 1. Prior to conducting the study, we administered an online questionnaire survey among the general public, asking them to select the most relaxing music from five pieces of music. These musical compositions were carefully selected from relaxing music pool available in the Guided and Imagery Music course, offered by the Australian Music and Imagination Society, a highly esteemed association in Australia. The criteria for defining relaxing music were as follows: (1) the melodic line exhibites a predictable pattern and is characterized by repetition; (2) the harmonic aspect remains tonal and consonant. (3) the music features predominantly legato phrasing with minimal dynamic variations; (4) the tempo remains steady, consistent and slow. Based on the voting results, it was determined that *Scent Of A Morning* by *Daydream* emerged as the relaxing music, whose execution time was approximately 4 min. The relaxing music was played through loudspeakers in our research, considering its universal characteristics. Their preferred music was collected either by their legally authorized representatives or directly from the controls. The preferred music was played live by a trained music therapist, who sat in front of the participants and played the piano while monitoring their blood pressure and heart rate using a pulse oximeter. This allowed the music to be adjusted based on participants’ physiological responses. The healthy controls were instructed to keep their eyes open, keep salient, and pay as much attention to music as possible. Additionally, we administered the standard arousal facilitation protocol outlined by the CRS-R guideline to ensure wakefulness in patients with DoC. Furthermore, for each participant, the auditory volume was maintained above their auditory threshold as assessed prior to EEG recording by BAEP.

### 2.3 EEG recording and audio recording

A Neuroscan system (Compumedics, United States) with a 64-channel amplifier (SynAmp2) following the international 10–20 system, was used to record electrophysiological activity. Data were sampled online at 2000 Hz with a 50 Hz notch filter. Impedance was kept below 10 kΩ.

A computer sound card, equipped with a sampling rate of 44,100 Hz, was used to record auditory signals. Additionally, a specially designed sync signal, used to align the EEG signals and auditory signals, was simultaneously recorded on the Neuroscan system and the sound card.

### 2.4 Aligning signals

All offline analyses were conducted using custom-written scripts and EEGLAB (14.1.1b) in MATLAB (version 2019a, MathWorks Inc., Natick, Massachusetts, USA). After listening to the auditory recording, the start and end points of each music piece were manually marked, and then the EEG data and audio signals were aligned with the synchronization signal through a cross-correlation function. The synchronization delay was controlled within 0.5 milliseconds. Therefore, there were three different music phases for each individual, including BL, RM and PM.

### 2.5 EEG preprocessing

First, each music stage underwent a low-pass filtering at 0.5 Hz and subsequently a high-pass filtering at 45 Hz using a Kaiserwin window sinc Finite Impulse Response (FIR) filter. Second, according to visual inspection, noisy data were removed and any channels identified as bad channels were manually interpolated. Third, to further eliminate nonlinearity artifacts, artifact subspace reconstruction (ASR) was performed by setting the cutoff parameter at 20, which has been verified to effectively remove nonbrain artifacts while preserving real brain signals (Chang et al., 2020). Fourth, Due to eye and muscle artifacts, components stemming were excluded by means of independent components analysis (ICA). Finally, the data were resampled to 500 Hz. We focused on the consciousness-related network, i.e., the frontal–parietal network. The relevant channels reflecting the frontal–parietal network were selected according to a previous study (Hinterberger et al., 2011). The targeted channels included FP1, FP2, AF3, AF4, Fz, F3, F4, F5, F6, F7, F8, Pz, P1, P2, P3, P4, P5, P6, P7, P8, POZ, PO3, PO4, PO7, and PO8.

### 2.6 Cerebral-acoustic coherence analysis

Cerebral-acoustic coherence (CACoh) (Peelle et al., 2013) was calculated to investigate the synchronization between the music envelope and the corresponding EEG data. For the BL and RM stages, the music envelopes were filtered using the same filter the EEG data and then resampled to 500 Hz. Both the EEG data and the music envelope were filtered between 1 Hz and 20 Hz (step: 0.5 Hz, bandwidth: 1 Hz) using the pop_eegfiltnew function in MATLAB. The Hilbert transform was used to extract the phase information. The neural response and the acoustic envelope were then segmented into zero-padded 5 s per trial. The coherence between the single-trial envelope and the EEG was quantified in each frequency bin (Harding et al., 2019). For each participant, the mean values of CACoh were computed across trials and interested channels.

Given that previous researches have observed stronger neural synchronization in musical pulse frequency and its harmonics frequencies (Tierney and Kraus, 2015; Tichko et al., 2022), and considering the differences in preferred music in our study, we focused on analyzing the neural entrainment to both the pulse frequency and its corresponding harmonic frequency of individualized music. Initially, we identified the pulse frequency of individualized music by extracting the peak frequency from the power spectrum of the music envelope. We then calculated the corresponding CACoh value for both the pulse frequency and its corresponding harmonic frequency over each individualized music.

Given the varying epoch numbers in different music stages, a total of 42 epochs were randomly selected for each music stage, based on the minimum epoch number. This randomization procedure was repeated 200 times. Subsequently, we obtained the mean CACoh value of the repeated BL, RM and PM separately. To estimate whether the observed CACoh of RM or PM was significantly distinguished from chance, we performed a paired samples t-test between BL and RM or PM for each group.

### 2.7 Phase-amplitude coupling analysis

To accurately capture the coupling relationship, it was crucial that the phase filter to have a narrow bandwidth, while the amplitude filter should have a bandwidth that was at least twice as wide as the upper limit of the phase filter (Dvorak and Fenton, 2014). Therefore, the phase series were filtered in the range from 1 Hz to 8 Hz (step: 1 Hz; bandwidth: 1 Hz), and the center frequency of the amplitude series was filtered in the range from 8 Hz to 40 Hz (below 30 Hz in 2 Hz steps, others in 5 Hz steps, both with specific bandwidths). The filtering process was performed using a zero-phase windowed sinc FIR filter. Phase and amplitude were then extracted from these filtered series using the Hilbert transform. For each stage, EEG data were divided into 5-s epochs without overlap, and the redundant data on both sides were eliminated. The modulation index (MI) method was employed to calculate the phase-amplitude coupling (Tort et al., 2010).

To determine whether the raw MI was independent of chance, a surrogate method was used to calculate the normalized MI (MIzscore) (Aru, 2015). Surrogate data were generated by segmenting the amplitude time series into ten sections and then randomly assembling them. Subsequently, we calculated 200 surrogate MI values for each epoch of each frequency pair. The MIzscore was obtained by subtracting the mean of 200 surrogate MI values and then dividing by the standard deviation. Only if the MIzscore exceeded 1.64, corresponding to a significance level of 0.05, was the corresponding MI reserved for the following calculation.

The repeated random sampling procedure was carried out the same manner as described above. We then averaged the significant MI value related to the target frequency pair, encompassing delta/theta-alpha/beta/gamma phase-amplitude coupling.

### 2.8 Statistical analysis

We used a one-way analysis of variance (ANOVA) with group (*VS*/UWS, MCS and HCs) as the independent variable, to determine whether the phase entrainment to music differed among groups. A one-way repeated measures ANOVA was performed to examine changes in MI strength after music performance. The between-group factor was the group (*VS*/UWS, MCS and HCs) and the within-group factor was the music condition (BL, RM and PM). Where there was a statistically significant interaction, *post hoc* correction was performed for multiple comparisons was performed. For subgroup analysis of MCS patients, another one-way repeated measures ANOVA was performed with the group (MCS_P and MCS_N) as the between-group factor when the within-group factor was the same as the previous one. Additionally, correlation analysis was performed using the Pearson correlation coefficient to evaluate the relationship between the MI change ratio of PM vs. BL and the predicted positive outcome rate. Outliers were removed prior to each analysis. *p* < 0.05 was considered as the significance threshold. All statistical analyses were conducted using SPSS (Version 25).

## 3 Results

### 3.1 Demographic and clinical data

Tables 1, 2 show the demographic and clinical data, respectively. There were no significant differences in the age and gender distributions among the *VS*/UWS, MCS, and HC groups (*F*_(2,44)_ = 2.579, *p* = 0.087; χ^2^(2) = 2.058, *p* = 0.357). The MCS group did not differ from the *VS*/UWS group by in terms of time since injury (*t*_(29)_ = 0.192, *p* = 0.849). However, the MCS group demonstrated a significant difference from the *VS*/UWS group in etiology (Fisher’s exact test: *p* = 0.023).

**Table 1:** The demographic information of healthy controls and patients.

	*VS*/UWS	MCS	HCs	Effect size	*p*-value
Age	44.5 ± 12.3	44.8 ± 13.8	36.1 ± 10.3	*F*_(2,44)_ = 2.579	0.087^a^
Gender (Male/Female)	11/5	8/7	7/9	χ^2^(2) = 2.058	0.357^b^
Etiology (TBI/nTBI)	2/14	8/7	–		0.023^c^
Months since injury	4.1 ± 3.0	3.9 ± 2.5	–	*t*_(29)_ = 0.192	0.849^d^

VS/UWS, vegetative state/unresponsive wakefulness syndrome; MCS, minimally conscious state; HCs, healthy controls; TBI, traumatic brain injury; nTBI, non-traumatic brain injury. The significance threshold was set at *p* < 0.05.

a One-way ANOVA.

b Chi-square test.

c Fisher’s exact test.

d Independent samples *t*-test.

**Table 2:** Clinical data of the patients.

Patient	Age/Gender	Etiology	Months since injury	CRS-R scores	Outcomes in 3 months (CRS-R scores)
*VS*/UWS1	44/M	TBI	4	1–0–2-1-0-1	1–0–2-1-0-2
*VS*/UWS2	22/M	TBI	4	1–0–2-1-0-2	1–0–2-2-0-2
*VS*/UWS3 *	50/M	nTBI	2	0–0–2-0-0-2	2–3–5-2-0-2
*VS*/UWS4	37/M	nTBI	2	1–0–2-1-0-2	1–0–2-1-0-2
*VS*/UWS5	50/M	nTBI	1	1–0–2-1-0-2	0–0–2-2-0-2
*VS*/UWS6	44/M	nTBI	6	1–0–2-1-0-0	0–0–2-2-0-0
*VS*/UWS7	37/F	nTBI	12	1–0–2-1-0-2	1–1–2-1-0-2
*VS*/UWS8	48/M	nTBI	2	1–1–2-1-0-2	1–0–2-1-0-2
*VS*/UWS9	47/M	nTBI	9	1–1–2-1-0-2	1–1–2-2-0-2
*VS*/UWS10	60/M	nTBI	1	0–1–2-1-0-2	1–1–2-2-0-2
*VS*/UWS11	65/F	nTBI	5	1–0–2-2-0-2	Death
*VS*/UWS12	35/M	nTBI	4	2–0–2-1-0-2	2–1–2-2-0-2
*VS*/UWS13	30/M	nTBI	2	1–0–2-1-0-2	1–0–2-1-0-2
*VS*/UWS14	55/F	nTBI	3	1–0–2-2-0-2	1–1–2-2-0-2
*VS*/UWS15	28/F	nTBI	3	2–0–2-2-0-2	1–1–2-1-0-2
*VS*/UWS16	60/F	nTBI	6	1–0–2-1-0-2	0–0–2-1-0-2
MCS1 *	57/M	TBI	8	3–5–5-2-1-2	4–5–5-2-2-2
MCS2 *	30/F	TBI	3	0–3–2-1-0-2	4–5–6-0-1-2
MCS3	24/M	TBI	3	1–3–2-1-0-2	2–3–2-2-0-2
MCS4	40/M	nTBI	1	1–1–5-1-0-2	1–1–5-2-0-2
MCS5 *	26/M	TBI	1	0–0–5-1-0-2	4–5–6-3-2-3
MCS6	47/F	nTBI	7	1–1–5-1-0-2	1–1–5-2-0-2
MCS7	52/F	nTBI	1	4–5–0-1-1-2	4–5–0-2-1-2
MCS8	65/M	nTBI	9	1–4–5-1-0-2	1–4–5-1-0-2
MCS9 *	50/M	nTBI	2	2–3–5-1-0-2	2–3–6-1-0-2
MCS10 *	63/F	TBI	2	2–3–5-2-0-2	3–4–6-2-0-2
MCS11 *	40/M	nTBI	5	1–4–5-1-0-2	1–4–6-2-0-2
MCS12	33/F	TBI	4	0–1–3-2-0-2	1–1–3-2-0-2
MCS13 *	60/F	nTBI	4	2–1–3-1-0-2	3–4–5-1-0-2
MCS14	54/F	TBI	5	1–3–3-1-0-2	1–3–2-1-0-2
MCS15 *	31/M	TBI	4	2–3–5-2-0-2	4–5–6-2-0-2

VS/UWS, vegetative state/unresponsive wakefulness syndrome; MCS, minimally conscious state; MCS-, minimally conscious state-; MCS+, minimally conscious state+; EMCS, emergence from minimally conscious state; TBI, traumatic brain injury; nTBI, non-traumatic brain injury; CRS-R, Coma Recovery Scale-Revised. * indicates better outcome 3 months later.

Outcome data were available for all patients. The MCS patients were categorized into two groups based on their three-month outcomes, with 8 patients showing positive outcomes and 7 patients exhibiting negative outcomes. We did not include the *VS*/UWS group as there was only one patient with a good outcome in this study.

### 3.2 Cerebral-acoustic coherence analysis

**Figure 2:**
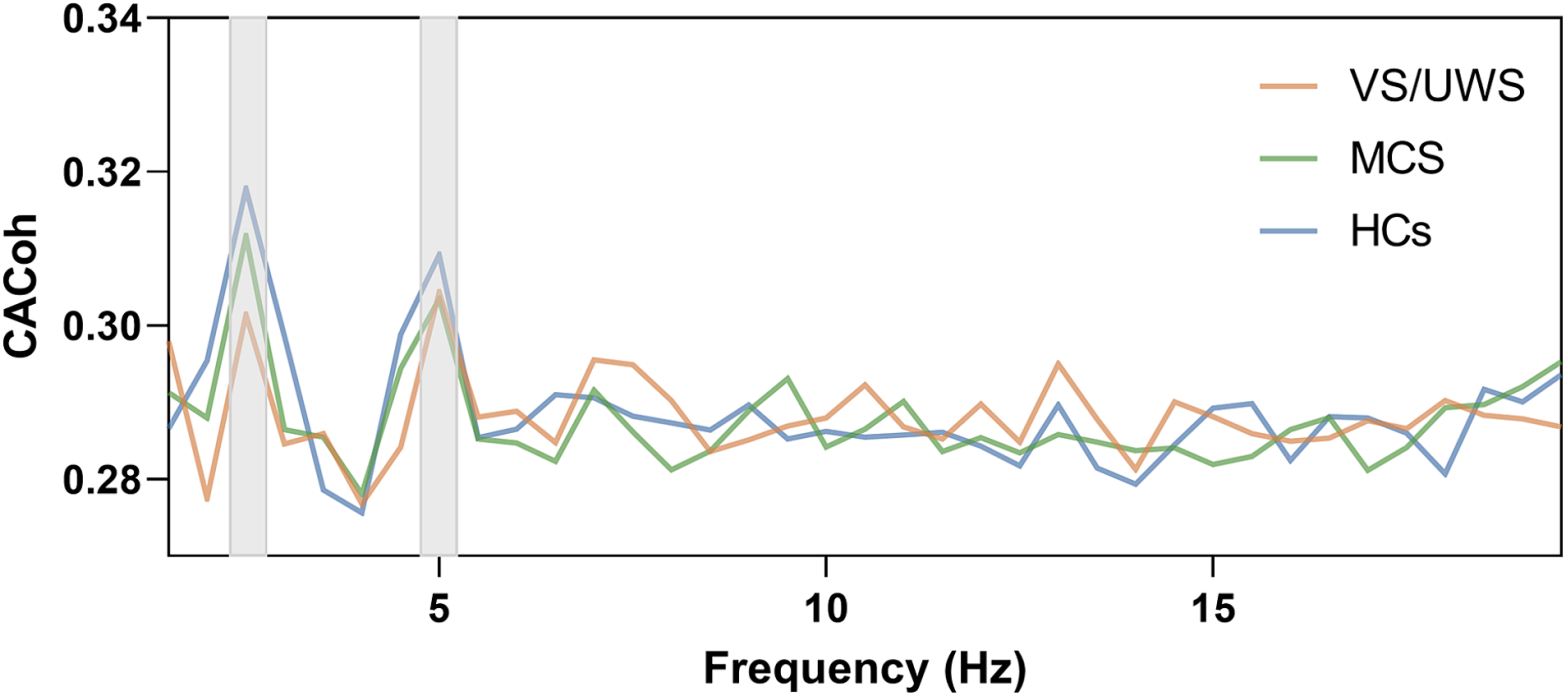
Neural entrainment to music in the HCs group, MCS group and *VS*/UWS group. Cerebral-acoustic Coherence (CACoh) spectrum to RM among HCs, MCS and *VS*/UWS groups. Significant coherences were present across the three groups in delta (2–3 Hz) band (*t*_(14)_ = 3.974, *p* < 0.001; *t*_(13)_ = 5.909, *p* < 0.001; *t*_(15)_ = 4.740, *p* < 0.001, for the three groups respectively) and theta (4.5–5.5 Hz) band (*t*_(14)_ = 2.737, *p* = 0.008; *t*_(13)_ = 1.842, *p* = 0.044; *t*_(15)_ = 4.761, *p* < 0.001, for the three groups respectively).

**Figure 3:**
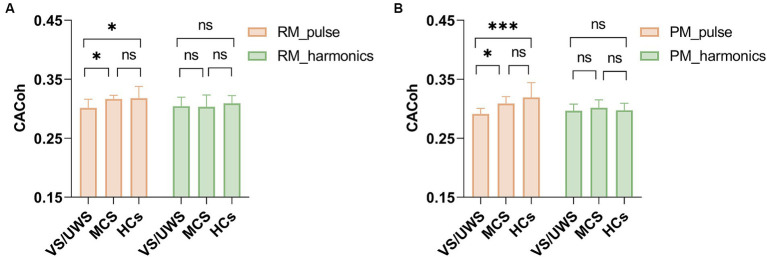
CACoh in the RM and PM conditions among HCs, MCS and *VS*/UWS groups. **(A)** The CACoh of musical pulse frequency was significantly different among the three groups in RM (*F*_(2, 40)_ = 5.201, *p* < 0.01), with higher CACoh in HCs and MCS than in VS/UWS (*p* = 0.016, *p* = 0.045, after Bonferroni correction). **(B)** The CACoh of musical pulse frequency was significantly different among the three groups in PM (*F*_(2, 39)_ = 8.886, *p* < 0.001), with higher CACoh in HCs and MCS than in *VS*/UWS (*p* < 0.001, *p* = 0.048, after Bonferroni correction). There were no significant differences among the three groups in harmonics frequency CACoh of RM and PM conditions. Abbreviations: RM, relaxing music; *VS*/UWS, vegetative state/unresponsive wakefulness; MCS, minimally conscious state; HCs, healthy controls; CACoh, cerebral-acoustic coherence; PM, preferred music. ^*^p < 0.05, ^***^p < 0.001, ^ns^ no significance.

To determine whether successful neural entrainment was achieved during music listening and to which frequency band it was achieved, we compared the observed CACoh value of RM with the CACoh value of BL per group and per frequency bin, as RM here was kept consistent across patients. We found that significant phase-locked neural responses to music were present in HCs, MCS patients and *VS*/UWS patients. These responses appeared mainly in the delta (2–3 Hz) band (*t*_(14)_ = 3.974, *p* < 0.001; *t*_(13)_ = 5.909, *p* < 0.001; *t*_(15)_ = 4.740, *p* < 0.001, for the three groups respectively), the pulse frequency of RM, and the theta (4.5–5.5 Hz) band (*t*_(14)_ = 2.737, *p* = 0.008; *t*_(13)_ = 1.842, *p* = 0.044; *t*_(15)_ = 4.761, *p* < 0.001, for the three groups respectively), the harmonic frequency of pulse frequency (Figure 2). In addition, one-way ANOVA analysis revealed a significant difference among the three groups (*F*_(2, 40)_ = 5.201, *p* < 0.01) at the RM pulse level. Compared to the *VS*/UWS group, both the HCs (*p* = 0.016) and MCS (*p* = 0.045) groups exhibited stronger coherence (Figure 3A). In the PM condition, all three groups showed significant entrainment of both musical pulse (*t*_(14)_ = 2.400, *p* = 0.015; *t*_(13)_ = 3.100, *p* = 0.004; *t*_(15)_ = 4.047, *p* < 0.001, for the three groups respectively) and harmonics frequency (*t*_(14)_ = 2.514, *p* = 0.012; *t*_(13)_ = 2.217, *p* = 0.023; *t*_(15)_ = 2.430, *p* = 0.014, for the three groups respectively). Moreover, there was a significant difference among the three group at the PM pulse level (*F*_(2, 39)_ = 8.886, *p* < 0.001) with the HCs group (*p* < 0.001) and MCS group (*p* = 0.048, Figure 3B) demonstrated a higher level of CACoh than the *VS*/UWS group. There were no significant differences in the harmonic frequency among the three groups in either the RM or PM condition.

### 3.3 Phase-amplitude coupling analysis

#### 3.3.1 *VS*/UWS, and HCs: phase-amplitude coupling

**Figure 4:**
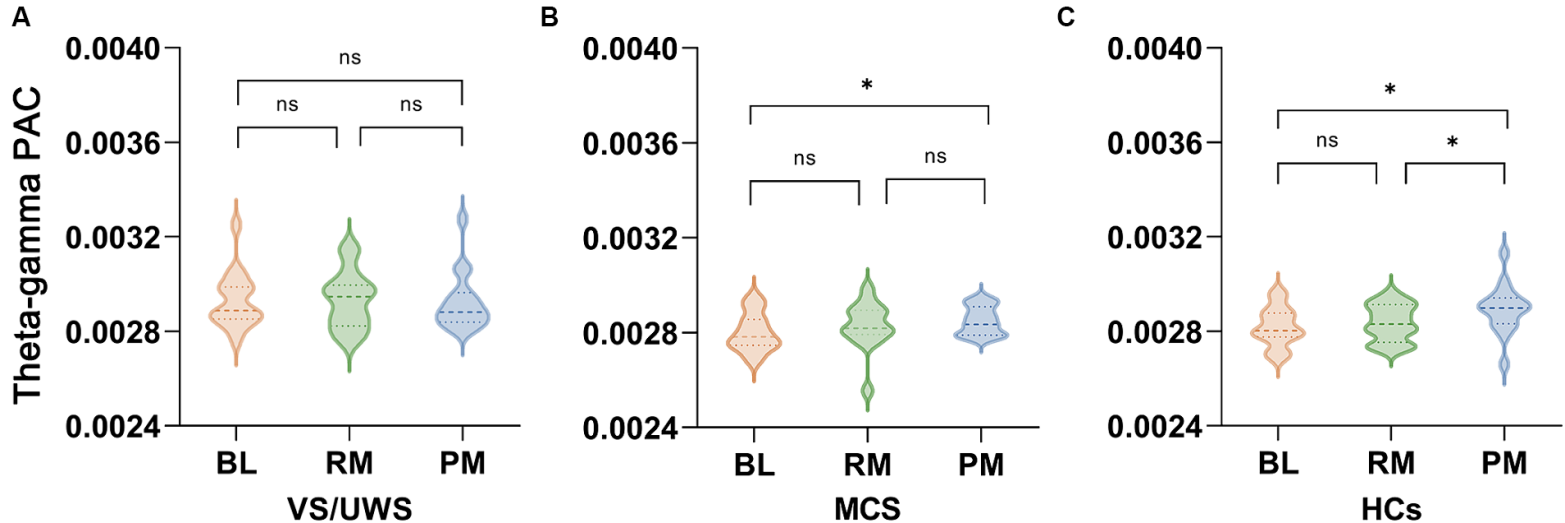
Music induced phase-amplitude coupling (PAC) in the *VS*/UWS patients, MCS patients and HCs. The theta-gamma PAC among the three groups. Repeated measures of ANOVA showed that only the theta-gamma pair exhibited significant interaction between group (*VS*/UWS, MCS and HCs) and music type (BL, RM and PM) (*F*_(2, 44)_ = 2.685, *p* = 0.036). **(A)** There was no significant difference among BL, RM and PM in *VS*/UWS patients. **(B)** Theta-gamma PAC was stronger in PM than in BL (*p* = 0.040, after Bonferroni correction). **(C)** PM induced marked coupling compared with BL and RM (*p* < 0.001, *p* = 0.016, after Bonferroni correction). Abbreviations: PAC, phase-amplitude coupling; BL, baseline; RM, relaxing music; PM, preferred music; *VS*/UWS, vegetative state/unresponsive wakefulness; MCS, minimally conscious state; HCs, healthy controls; * *p* < 0.05, ^ns^ no significance.

We conducted phase-amplitude coupling analysis on different frequency pairs in various music conditions to evaluate whether music could enhance communication within the frontal–parietal network. A significant interaction effect was observed solely in theta-gamma PAC (*F*_(2, 44)_ = 2.685, *p* = 0.036). *Post hoc* analysis revealed that PM exhibited stronger coupling than the RM (*p* = 0.016) and BL (*p* < 0.001) in the HCs group (Figure 4C). The coupling was stronger in the preferred music stage than at baseline in MCS patients (*p* = 0.040, Figure 4B), whereas there was no significant difference among the three music types in the *VS*/UWS group (Figure 4A).

#### 3.3.2 Patients with positive and negative outcomes: phase-amplitude coupling

**Figure 5:**
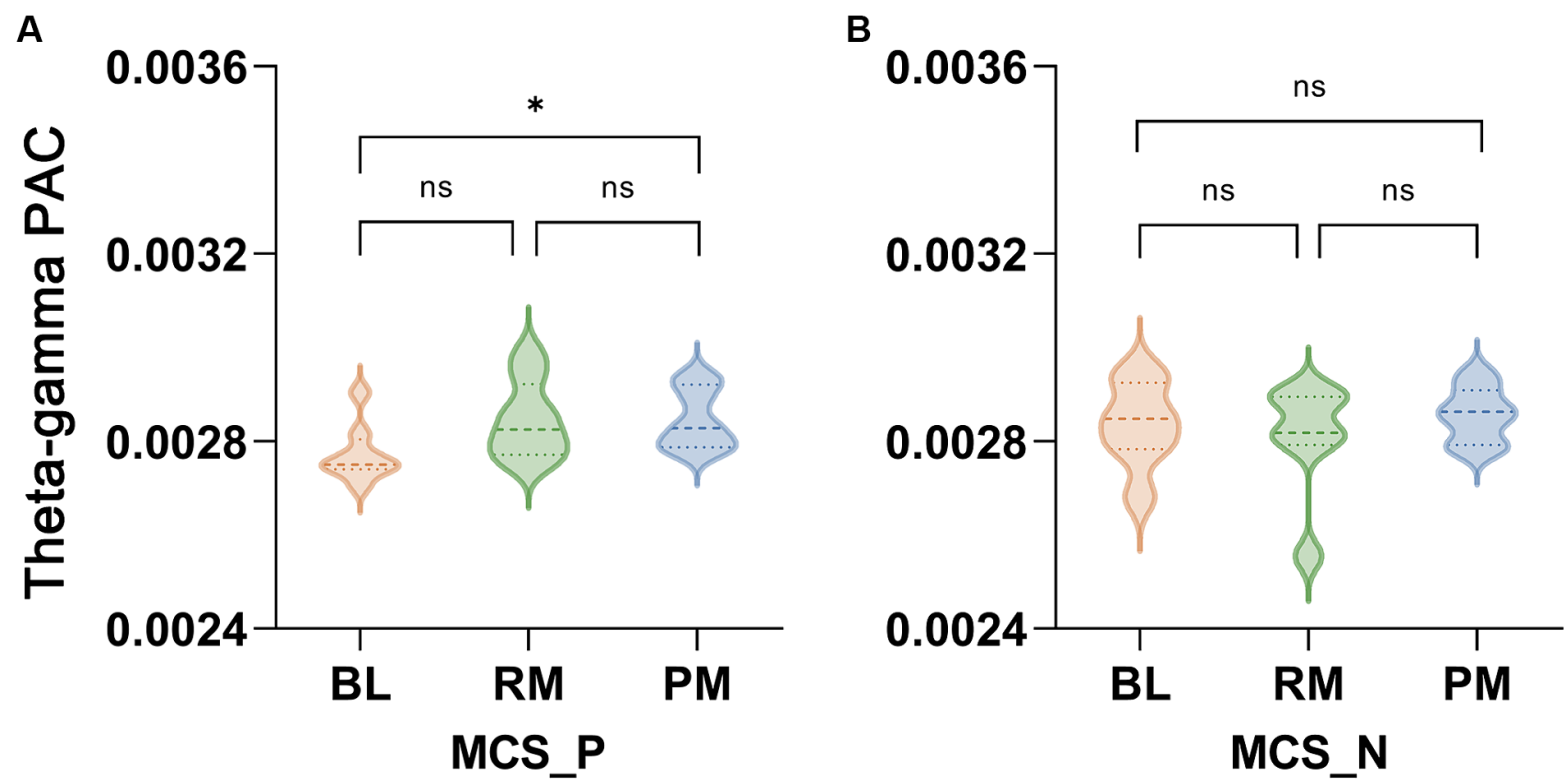
The theta-gamma PAC in the MCS_P and MCS_N groups. MCS patients were divided into two groups according to their three-month outcomes and patients with better outcomes were regarded as MCS_P while patients with bad outcomes were considered as MCS_N. A significant interaction between group (MCS_P and MCS_N) and music type (BL, RM and PM) was found in theta-gamma PAC (*F*_(2, 12)_ = 3.709, *p* = 0.038). **(A)** In the MCS_P group, there was significantly stronger coupling in PM than in BL (*p* = 0.019). **(B)** No significant difference was found in the MCS_N group. Abbreviations: PAC, phase-amplitude coupling; BL, baseline; RM, relaxing music; PM, preferred music; MCS_P, minimally conscious state-positive outcome; MCS_N, minimally conscious state-negative outcome; ^*^p < 0.05, ^ns^ no significance.

To investigate whether such stronger coupling in the PM stage was related to the outcomes of patients, we subsequently categorized the MCS patients into two subgroups based on their outcomes, the MCS_P group and MCS_N group. Notably, a significant interaction effect was observed in theta-gamma PAC (*F*_(2, 12)_ = 3.709, *p* = 0.038). Only MCS_P showed significantly enhanced coupling in PM compared to BL (*p* = 0.019, Figure 5).

#### 3.3.3 Correlation between phase-amplitude coupling and the outcomes of MCS patients

**Figure 6:**
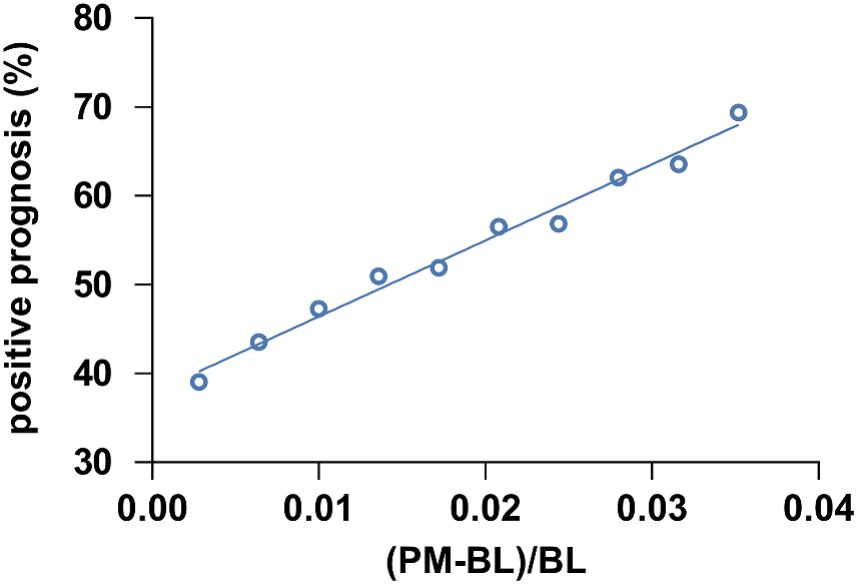
The correlation between the theta-gamma PAC ratio from BL to PM and the positive prognosis of patients with MCS. The theta-gamma PAC at (PM-BL)/BL was correlated with the positive prognosis of MCS patients (*r* = 0.992, *p* < 0.001). Abbreviations: (PM-BL)/BL, the thete-gamma phase-amplitude coupling change ratio from the baseline condition to the preferred music condition.

To further evaluate whether the ratio of theta-gamma phase-amplitude coupling changes between PM and BL could predict the outcomes in MCS patients, we performed a correlation analysis. We employed a bootstrap method where we calculated the ratio of theta-gamma PAC strength changes of PM relative to BL and pooled all ratio data from MCS patients. Subsequently, a subset of seven data points was randomly selected to match the number of MCS_N cases. This procedure was repeated 1,000 times to obtain mean coupling change ratios and the positive outcome rate for each repetition. The outcome rates were then sorted into 10 bins and the average coupling change ratio was computed for each respective bin. A significant correlation was observed between the ratio of coupling change ratio and the positive outcome rate, as revealed by a Pearson correlation analysis (*r* = 0.992, *p* < 0.001, Figure 6). In addition, we conducted a correlation between the ratio of coupling change and the post-injury time. However, there were no significant correlations (*r* = 0.038, *p* = 0.892).

## 4 Discussion

The main objectives of this research were to determine: (1) whether patients with DoC could neutrally entrain to music, (2) whether cross-modal influences contribute to the improvement of consciousness level when music works on patients with DoC, based on phase-amplitude coupling, (3) whether individualized music works more efficiently than relaxing music and whether the cross-modal influences could determine the outcomes of patients. Our findings demonstrated that both healthy participants and patients with DoC exhibited neural entrainment to music, with HCs and MCS patients showing stronger CACoh than *VS*/UWS patients. We also found that both healthy participants and MCS patients exhibited enhanced theta-gamma PAC within the frontal–parietal network during the individualized music condition compared to BL. Furthermore, those MCS patients who showed positive outcomes at 3 months demonstrated an evident response to music, as reflected by increased theta-gamma phase-amplitude coupling strength in the PM condition. Additionally, the ratio of changes in theta-gamma coupling in PM relative to BL could forecast clinical outcome in MCS patients. In summary, our findings suggest that phase-amplitude coupling may serve as a marker of musical cross-modal influences in patients with DoC and individualized music may be a more forward-oriented rehabilitation method for patients with DoC.

Our study demonstrated that not only HCs but also patients with DoC exhibit neural entrainment to music, with significant cortical tracking observed in the delta and theta frequency bands, suggesting that both of them are capable of perceiving music (Doelling and Poeppel, 2015; Obleser and Kayser, 2019). Our results are consistent with a previous study which found that the ongoing oscillations in patients with DoC can be phase-locked to neutral speech and modulated tones (Xu et al., 2021). The ongoing neural oscillations gradually synchronize with the musical beat or meter, realizing the perception of musical rhythm, which is fundamental to music perception. Neural entrainment has been proposed as the neural correlate underlying the perception of musical rhythm (Large et al., 2015; Weineck et al., 2022). Neural entrainment reflects the selective attention to the external environment and ensures optimal event processing (Helfrich et al., 2019; Obleser and Kayser, 2019; Kachlicka et al., 2022). In line with our expectations, patients with DoC demonstrated the ability to track music, indicating their preserved cortical ability to track music. Furthermore, we observed that both HCs and MCS patients showed a stronger CACoh than *VS*/UWS patients, suggesting a reduced perception of external stimuli in *VS*/UWS patients. Consequently, neural entrainment to music holds promise as a potential method for distinguishing MCS patients from *VS*/UWS patients.

We found that musical cross-modal influences on consciousness were based on phase-amplitude coupling within the frontal–parietal network. During preferred music, there was an enhancement of theta-gamma PAC within the frontal–parietal network, particularly observed in those who had better three-month outcomes. This result suggests that the change of the theta-gamma PAC strength from baseline to individualized music could serve as a predictive indicator of the patients outcomes. Several studies have demonstrated disrupted connections within the frontal–parietal cortex in patients with DoC (Cauda et al., 2008; Wu et al., 2020; Panda et al., 2022). Phase-amplitude coupling has been demonstrated to play a functional role in information transfer within the brain (Hyafil et al., 2015; González et al., 2020). This result indicates that by enhancing theta-gamma PAC within the frontal–parietal network, that is, by facilitating information integration and neural processing within the consciousness-related network, music may ameliorate broken brain connections and promote recovery of consciousness. In this context, cross-modal influences play a crucial role in conscious restoration. Our findings indicate that phase-amplitude coupling within the frontal–parietal network may serve as an indicator of musical cross-modal influences on consciousness.

In our study, theta-gamma phase-amplitude coupling was enhanced after music. Phase-amplitude coupling has been hypothesized to play a crucial role in the temporal parsing of (quasi) rhythmic stimulation (Hyafil et al., 2015). Previous studies suggest an association between theta-gamma PAC and auditory processing, with increased coupling when the processing performs better (Papadaniil et al., 2015; Mai et al., 2016; Tseng et al., 2019; Huang et al., 2021). This is argued that theta oscillations in the cortices reflect the rhythm of simulation, whereas gamma oscillations represent the higher-level processing. Musical rhythms occur in a particular frequency range corresponding to the delta and theta frequency bands (London, 2004; Large et al., 2015), the perception of which can be achieved through the entrainment of delta and theta endogenous oscillations in the brain during music listening. Low-frequency entrainment offers a method for temporal integration of information and selective attention (Lakatos et al., 2008, 2019; Kachlicka et al., 2022; Nandi et al., 2023), while gamma oscillations are related to predictive mechanisms and underpin fine-grained sensory processing (Fries et al., 2007; Fries, 2009; Herff et al., 2020; Parto-Dezfouli et al., 2023). For example, a previous study reported that the gamma oscillation predicted the next tone onset (Snyder and Large, 2005). In essence, theta oscillations in cortices reflect the context of music, whereas gamma oscillations represent the processing of musical content. Theta frequency coupled with gamma frequency facilitates the perception and processing of music.

Moreover, we observed that HCs and MCS patients exhibited enhanced theta-gamma PAC exclusively during the preferred music condition. Our findings are accordant with a previous study that demonstrated a markedly enhanced level of responsiveness to preferred music, as compared to neural stimuli (Heine et al., 2017). This may be attributed to the fact that preferred music has the ability to evoke unique emotional experiences in each individual (Vuilleumier and Trost, 2015; Luauté et al., 2018). Patients with DoC are more likely to be attracted to sensory stimulation that have an emotional component (Gao et al., 2019), making it likely that emotional music may elicit broader and stronger brain activation. In addition, individualized music has been described as self-referential and familiar music that may evoke memories associated with specific musical experiences (Kaiser and Berntsen, 2023). Previous studies have shown enhanced connections between the dorsal medial prefrontal cortex (MPFC) and the external network (Janata, 2009) when we recall autobiographical memories through familiar songs. The MPFC, a constituent of the internal network, is considered a fundamental element of the cerebral cortex related to consciousness on the basis of GWT (Dehaene and Changeux, 2011; Seth and Bayne, 2022). In other words, individualized music boosts communication within the consciousness-related network. Consequently, our results suggest that preferred music may provide a restorative mechanism for patients with DoC when compared to relaxing music.

The results of our study revealed no statistically significant distinction in the theta-gamma PAC strength between relaxing music and baseline across all three groups. However, a previous study demonstrated that the utilization of recorded relaxing music can effectively contribute to patient’s relaxation and levels of consciousness, as it has been shown to reduce blood pressure and increase oxygen saturation (Ribeiro et al., 2014). Relaxing music possesses the ability to elicit a state of relaxation, thereby improving the function of the autonomic nervous system (Vigo et al., 2014). From this perspective, relaxing music may serve as a therapeutic intervention in facilitating the recovery for patients with DoC. The possibility exists that relaxing music presented in a live performance may elicit a more effective response. Therefore, it is considered that future studies should adopt a consistent live presentation of music to enable more accurate comparisons between the therapeutic effects of relaxing music and preferred music.

Our findings indicated that there was no discernible difference among the three music conditions in *VS*/UWS patients. This maybe because that patients with *VS*/UWS had more severe brain injury so that their ability to integrate information across brain was worse. Rizkallah et al. (2019) found that the connection to the left precuneus, which was engaged in self-related and episodic memory, was worst in patients with *VS*/UWS. Due to disrupted connection within the brain, patients with *VS*/UWS could not recall memories related to individualized music, and then the strength of theta-gamma PAC did not change significantly. Specifically, in this study, the majority of *VS*/UWS patients enrolled in this study were unconscious due to non-traumatic brain injury (nTBI), which is associated with more severe cortical damage in comparison with traumatic brain injury (TBI), especially hypoxic–ischemic encephalopathy (HIE) (Geocadin et al., 2019). This may explain why *VS*/UWS patients show no observable changes under music conditions here. Our study implies that musical cross-modal influences on patients with DoC may be affected by etiology. Our result also revealed that the increased theta-gamma PAC during PM condition did not exhibit any correlation with the duration of loss of consciousness. However, there is a phase of spontaneous evolution of the consciousness level in the first 2–6 months and early intervention in patients with DoC yields greater benefit in terms of improving their level of consciousness (Giacino et al., 2020 Ma et al., 2023). The discrepancy may be attributed to the fact that the majority of patients in our study were enrolled within 6 months, which corresponds to a relatively favorable recovery period, as the recovery duration of patients with DoC generally ranges from 1.5 to 12 months after injury (Giacino et al., 2012).

However, the current study encountered certain limitations. The result may be impacted by the etiology of patients with DOC. Future studies could consider increasing the sample size and then categorizing patients with DoC based on different etiologies as well as varying duration of onset, in order to provide a more comprehensive understanding of the therapeutic effects of music. In addition, it is possible that the duration of follow-up was insufficient, resulting in only one *VS*/UWS patient showing improvement in this study. Future investigation should extend the follow-up period to 1 year or longer to obtain a more accurate comprehension of the correlation between the predictive ability of music-induced phase-amplitude coupling caused by music and clinical outcomes. Our study only took the frontal–parietal network into account, and further studies could explore the effect of music on other brain regions. Also, the variations in music presentation may impact the findings, and subsequent studies can adopt a consistent approach to music presentation in order to enhance the result comparability and enable more accurate comparisons between the therapeutic effects of relaxing music and preferred music.

In conclusion, these findings suggest that patients with DoC could be neutrally entrained to music and that preferred music works as a potential method for patients with DoC through cross-modal influences, which depend on enhanced theta-gamma PAC within the consciousness-related network. Therefore, we propose that individualized music may potentially contribute to the recovery of consciousness and should be advocated in clinical practice for patients with DoC.

## Data availability statement

The original contributions presented in the study are included in the article/supplementary material, further inquiries can be directed to the corresponding authors.

## Ethics statement

The studies involving humans were approved by the Ethics Committee of Zhujiang Hospital. The studies were conducted in accordance with the local legislation and institutional requirements. The participants provided their written informed consent to participate in this study.

## Author contributions

QXia: Data curation, Writing – original draft. XZ: Data curation, Writing – review & editing. YW: Data curation, Writing – review & editing. ZY: Data curation, Writing – review & editing. ZC: Data curation, Writing – review & editing. YL: Data curation, Writing – review & editing. SL: Data curation, Writing – review & editing. XH: Validation, Writing – review & editing. HZ: Data curation, Writing – review & editing. CX: Writing – review & editing. CZ: Methodology, Software, Writing – review & editing. JP: Writing – review & editing. QXie: Funding acquisition, Writing – review & editing, Methodology, Validation.
